# Determining the SARS-CoV-2 serological immunoassay test performance indices based on the test results frequency distribution

**DOI:** 10.11613/BM.2022.020705

**Published:** 2022-06-15

**Authors:** Farrokh Habibzadeh, Parham Habibzadeh, Mahboobeh Yadollahie, Mohammad M. Sajadi

**Affiliations:** 1 Global Virus Network, Middle East Region, Shiraz, Iran; 2 Research Center for Health Sciences, Institute of Health, Shiraz University of Medical Sciences, Shiraz, Iran; 3 Freelance Researcher, Shiraz, Iran; 4 Institute of Human Virology, University of Maryland School of Medicine, Baltimore, USA; 5 Global Virus Network, Baltimore, USA

**Keywords:** COVID-19 testing, serologic tests, sensitivity, specificity, diagnostic tests

## Abstract

**Introduction:**

Coronavirus disease 2019 (COVID-19) is known to induce robust antibody response in most of the affected individuals. The objective of the study was to determine if we can harvest the test sensitivity and specificity of a commercial serologic immunoassay merely based on the frequency distribution of the SARS-CoV-2 immunoglobulin (Ig) G concentrations measured in a population-based seroprevalence study.

**Materials and methods:**

The current study was conducted on a subset of a previously published dataset from the canton of Geneva. Data were taken from two non-consecutive weeks (774 samples from May 4-9, and 658 from June 1-6, 2020). Assuming that the frequency distribution of the measured SARS-CoV-2 IgG is binormal (an educated guess), using a non-linear regression, we decomposed the distribution into its two Gaussian components. Based on the obtained regression coefficients, we calculated the prevalence of SARS-CoV-2 infection, the sensitivity and specificity, and the most appropriate cut-off value for the test. The obtained results were compared with those obtained from a validity study and a seroprevalence population-based study.

**Results:**

The model could predict more than 90% of the variance observed in the SARS-CoV-2 IgG distribution. The results derived from our model were in good agreement with the results obtained from the seroprevalence and validity studies. Altogether 138 of 1432 people had SARS-CoV-2 IgG ≥ 0.90, the cut-off value which maximized the Youden’s index. This translates into a true prevalence of 7.0% (95% confidence interval 5.4% to 8.6%), which is in keeping with the estimated prevalence of 7.7% derived from our model. Our model can provide the true prevalence.

**Conclusions:**

Having an educated guess about the distribution of test results, the test performance indices can be derived with acceptable accuracy merely based on the test results frequency distribution without the need for conducting a validity study and comparing the test results against a gold-standard test.

## Introduction

Serological tests are very helpful for sero-epidemiological studies. Coronavirus disease 2019 (COVID-19) is known to induce robust antibody response in most of the affected individuals (*1*). The antibody concentrations could serve as an important laboratory index with prognostic implications for patients recovering from COVID-19 (*2*). In spite of their limitations, serologic-based assays are currently the best available method to document past infections (*3*, *4*). Furthermore, they can be used to gauge individual’s immune response to severe acute respiratory syndrome coronavirus 2 (SARS-CoV-2) vaccines (*5*). However, despite the widespread applications of such assays, inter-individual variability in the immune response due to various factors such as host genetic build-up can have significant ramifications for the interpretations of such serologic assays (*6*, *7*). Therefore, identification of the performance indices of these assays could have wide-reaching applications in the correct interpretation of these tests, both in the clinical and public health contexts.

In a previous study, we have shown that it is possible to determine the performance indices of a diagnostic test with continuous results merely based on the frequency distribution of the test results in a population, without need for a gold standard test, by making an educated guess about the distribution (*8*). Application of this method to the distribution of prostate-specific antigen shows promising results. Herein, we apply the method to determine if we could harvest the indices solely based on the frequency distribution of SARS-CoV-2 immunoglobulin (Ig) G concentrations measured in a population-based study in Geneva, Switzerland, by a commercially available serological immunoassay and compare the results with those derived from a validity study conducted earlier (*9*, *10*).

## Materials and methods

### Study design

Data from a population-based seroprevalence study (SEROCoV-POP study) that measured anti-SARS-CoV-2 IgG antibodies in sera of the study participants from April 6 to May 9, 2020, in Geneva, Switzerland, using a commercially available ELISA test (Euroimmun; Lübeck, Germany, #EI 2606-9601 G) targeting the S1 domain of the spike protein of SARS-CoV-2, were used in the current investigation (*9*). The ELISA test performance had been evaluated in a case-control validation study on sera of 181 patients with confirmed SARS-CoV-2 and 326 pre-pandemic control serum samples against a whole spike protein-based recombinant immunofluorescence assay (rIFA, considered the gold-standard test) (*10*).

The final SEROCoV-POP study protocol and the detailed methodology are described elsewhere (*9*). In brief, the study was a population-based study performed on the former participants of the Bus Santé study and their household members. The Bus Santé study population included 20–74-year-old people identified through an annual residential list established by the local government (*11*). Permanent residents of institutions (*e.g.*, prisons and care homes) were excluded from the study (*9*).

About 1300 randomly selected individuals were selected weekly from the participants of the Bus Santé study and invited to participate along with all their household members aged ≥5 years in the SEROCoV-POP study. None of the participants had received a SARS-CoV-2 vaccine. All participants, regardless of their past history of COVID-19, were included in the study. Participants in quarantine or isolation or those with symptoms compatible with COVID-19 were asked to postpone their visit to a later date.

The current study was conducted on 2766 SEROCoV-POP study participants aged ≥5 years, selected from 1339 households – a representative sample of the canton of Geneva. A subset of the serological immunoassay data consisting of the blinded IgG data for two non-consecutive weeks (N = 1432 samples: 774 from May 4-9, and 658 from June 1-6, 2020) for individuals participating in this investigation conducted by *Unité d’Épidémiologie Populationnelle* of the *Hôpitaux Universitaires de Genève* (HUG) was used for our analyses.

### Educated guess

The ELISA test used in this study was designed to detect IgG antibodies against SARS-CoV-2. However, the similarity between some of the antigenic epitopes of the SARS-CoV-2 and other viruses (*e.g.*, HCoV-229E, -NL63, -OC43, *etc.*) caused cross-reactivity and false-positive test results (*10*). The frequency distribution of the measured antibodies might thus have two peaks – one for those with and another for people without SARS-CoV-2 antibodies. Despite the lack of a systematic framework to generate an educated guess, looking at the frequency distribution of the measured antibodies would give us a clue.

### Ethics

The protocol of the current study was approved by the Petroleum Industry Health Organization R&D Institutional Review Board. The protocol of the original seroprevalence study was approved by the Cantonal Research Ethics Commission of Geneva, Switzerland (*9*). All methods were performed in accordance with the relevant guidelines and regulations. Informed consent was obtained from all study subjects and/or their legal guardian(s) who had participated in the original seroprevalence study (*9*).

### Statistical analysis

**Figure 1:**
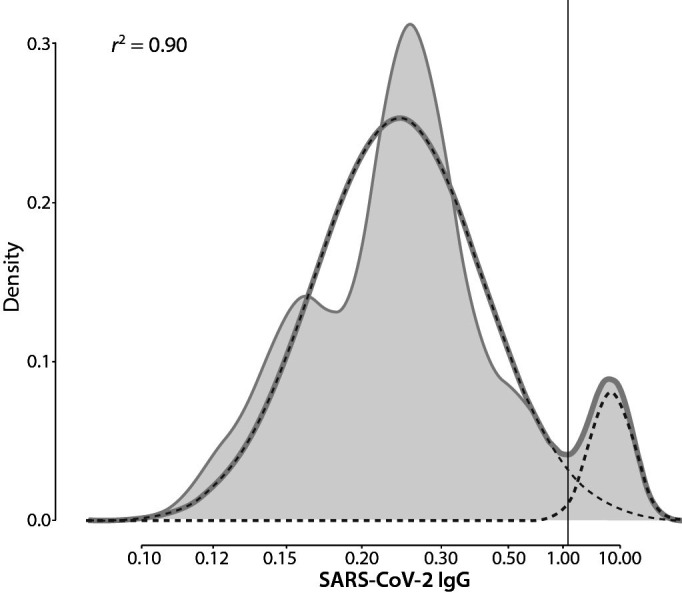
The relative frequency distribution of SARS-CoV-2 IgG (gray area). The thick gray curve is the binormal curve fitted to the data. The curve is in fact the result of superposition of two normal curves describing the relative frequency distribution of non-SARS-CoV-2 IgG antibodies (light gray dashed curve) and patients with SARS-CoV-2 IgG antibodies (dark gray dashed curve). The vertical black solid line represents the cut-off value. Note that the x-axis is not linear (transformed by a Box-Cox transformation with a λ of -0.869).

*R* software version 4.1.0 (2021-05-18) was used for data analysis. Box-Cox transformation was used to normalize the highly positively skewed IgG antibody frequency distribution (*12*). The *R* function *boxcox* of *EnvStats* package was used to optimize the transformation parameter (λ) using the log-likelihood function (*13*). Using the default values of the *R density* function, the density curve for the transformed IgG values was made. The function uses by default a Gaussian kernel, 512 bins, and a bandwidth calculated according to the Silverman’s rule (*14*). Using the density values, we then applied a binormal (based on our educated guess) non-linear regression, using the function *nlsLM* from *minpack.ml* package, as described earlier, to decompose the IgG frequency distribution into its two presumably normal components – the first component related to the distribution of antibody in those without SARS-CoV-2 IgG; the second, those with SARS-CoV-2 IgG (*8*). The general form of the binormal equation used for non-linear regression was:



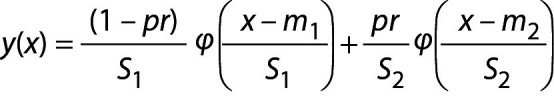



where *φ* is the probability density function of the normal (Gaussian) distribution; *x*, the transformed SARS-CoV-2 IgG concentration; *y*(*x*), the density function value at *x*; *pr*, the prevalence of SARS-CoV-2 infection; *m*_1_ and *m*_2_, means of the first and second normal components of the binormal curve (Figure 1); and *s*_1_ and *s*_2_, the standard deviations (*8*). The first and second terms in the above equation describe the frequency distribution of antibodies in those without and with SARS-CoV-2 IgG antibodies, respectively.

A receiver operating characteristic (ROC) curve was constructed based on the results obtained from the binormal model and compared with the ROC curve presented in the original validity study (*10*). Area under the ROC curve was calculated according to DeLong *et al.,* and compared to the area under the curve reported in the original validity study (*10*, *15*).

There are several criteria for determination of a test cut-off value. Although the most appropriate value could be determined by maximizing the weighted number needed to misdiagnose, we chose to maximize the Youden’s J (sensitivity + specificity – 1) to calculate the cut-off value since we had no idea about the cost (not limited to the financial aspects) of a false-negative test result relative to a false-positive result (*16*-*18*).

## Results

**Figure 2:**
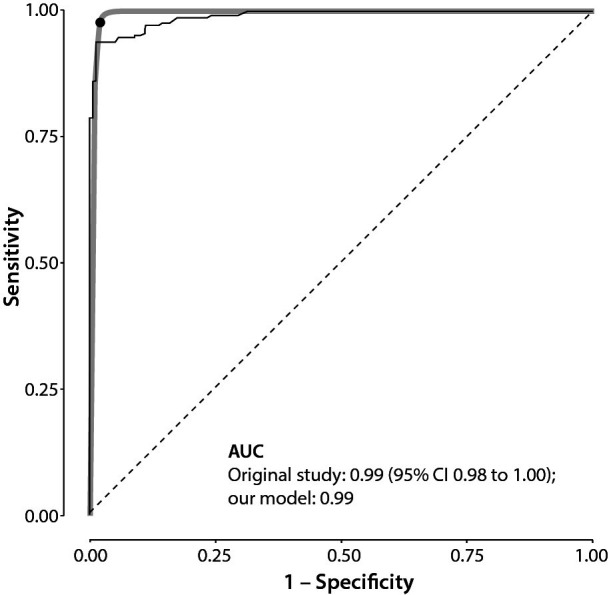
The receiver operating characteristic (ROC) curve for the test. The black curve is the one reported in Figure 1C of the original validity study (*10*). The gray curve was constructed based on the data obtained from our model. The 95% confidence interval (CI) of the area under the ROC curve (AUC) from the validity study includes the AUC derived by our model, 0.99. The red circle corresponds to the SARS-CoV-2 IgG cut-off value of 0.90.

**Figure 3:**
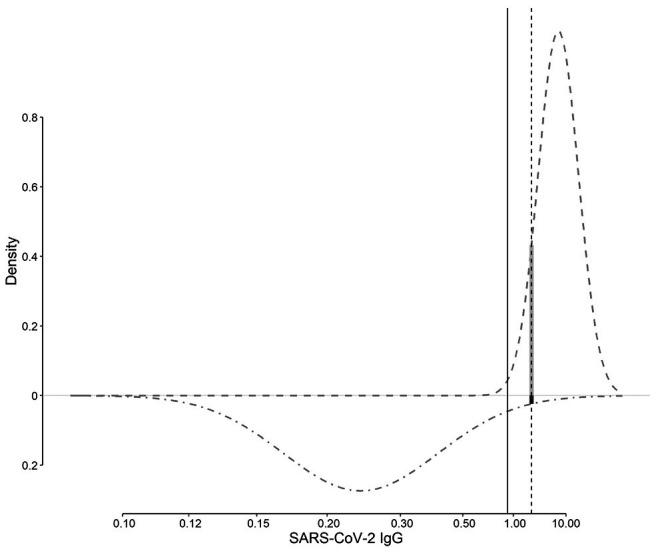
The density functions for the distribution of IgG in those with (dashed curve) and without SARS-CoV-2 IgG (dot-dashed curve). The vertical black solid line represents the cut-off value, 0.90. The two curves are density functions, which means the area under each curve is one. This implies that the function value at any given IgG value is equal to the probability of observing that IgG value in that group. For example, the probability of observing an IgG value of 1.5 (vertical dashed line) in a patient with SARS-CoV-2 is 0.432 (the height of the thick light gray bar) and 0.024 (the height of the thick dark gray bar) in those without SARS-CoV-2 antibodies. Note that the x-axis is not linear (transformed by a Box-Cox transformation with a λ of -0.869).

**Figure 4:**
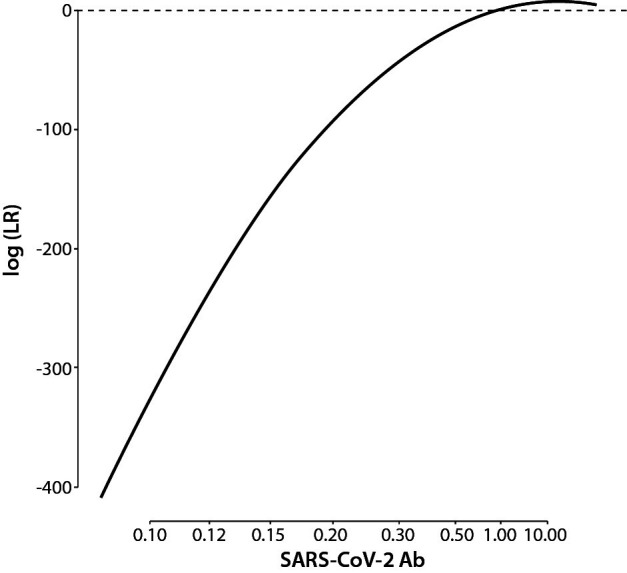
The likelihood ratio (LR) for each SARS-CoV-2 IgG antibody concentration. Note that the y-axis has a logarithmic scale (base 2) and that the x-axis is not linear (transformed by a Box-Cox transformation with a λ of -0.869). The LR varies from a minimum of 0 for very low values of IgG concentrations to a maximum of 127.33 at an IgG concentration of 70.84.

Sera of 1432 individuals – 758 (53%) females and 674 (47%) males, were studied. The participants had a median age of 49 (interquartile range (IQR) 31 to 60) years. The distribution of the measured SARS-CoV-2 IgG was highly skewed. The skewness decreased from 5.1 to 0.2 after a Box-Cox transformation (λ = -0.869). The binormal non-linear regression resulted in a good fit (*r*^2^ = 0.90, Figure 1). Our model revealed that the prevalence of those with SARS-CoV-2 IgG among the studied population was 7.7%; the most appropriate SARS-CoV-2 IgG cut-off value was 0.90, associated with a test sensitivity of 99% and a specificity of 97% (Figure 2). The ROC curve derived from our model overlapped with acceptable accuracy on the plot obtained from the validity study (Figure 2). Based on the results, we plotted the density functions for the distribution of IgG in those with and without SARS-CoV-2 IgG (Figure 3) as well as a plot showing the likelihood ratio corresponding to each given value of IgG (Figure 4).

Most serological tests are not perfect and may result in false-positive and false-negative results. Therefore, seropositive rate (apparent prevalence) is generally not equal to the prevalence of the disease of concern (true prevalence) (*19*, *20*). Based on the derived cut-off value, and test sensitivity and specificity, the apparent and true prevalence of the disease was calculated for the total data subset studied, as well as for each study week (Table 1).

**Table 1:** The apparent and true prevalence of the disease calculated based on the cut-off value of 0.90, test sensitivity of 99.4%, and test specificity of 97.1%, according to Rogan and Gladen (19)

**Data**	**N**	**Seropositive**	**Prevalence, % (95% CI)**	
			**Apparent**	**True**
May 4–9	774	89	11.5 (9.3 to 13.8)	8.9 (6.6 to 11.3)
June 1–6	658	49	7.5 (5.4 to 9.5)	4.7 (2.7 to 6.8)
Total	1432	138	9.6 (8.1 to 11.2)	7.0 (5.4 to 8.6)
CI - confidence interval.				

## Discussion

Diagnostics in general and serological tests are central and fundamental to quality health care and research. It has been shown that many serological tests used for the diagnosis of SARS-CoV-2 antibodies provide valid, consistent results (*21*-*23*). For instance, it has been shown that the results of MAGLUMI 2019-nCoV IgM and IgG (SNIBE, Shenzhen, China) are well aligned with those of Euroimmun anti-SARS-CoV-2 IgG and IgA (Euroimmun AG, Lüebeck, Germany), and that both immunochromatographic rapid IgM and IgG test and the chemiluminescence IgM and IgG immunoassay are useful tools for epidemiologic surveillance (*21*, *22*). However, none of the serological tests is perfect. To determine the test sensitivities and specificities, we need to compare them against a gold-standard, such as reverse-transcription polymerase chain reaction (RT-PCR) tests. Herein, we presented a technique based on which all the test performance indices (plus the prevalence of the disease of concern, as well as the most appropriate test cut-off value) can solely be computed based on the frequency distribution of the serological test values in a representative sample of a population, without the need for a gold standard.

The method we proposed provided results with an acceptable accuracy. The model predicted more than 90% of the variance observed in the SARS-CoV-2 IgG distribution. The most appropriate cut-off value of 0.90 we derived corresponds to the maximum Youden’s J index. However, there is no restrict rule for the determination of a test cut-off value. For example, if we want to have a more specific test to decrease the false-positive rate, we need to increase the cut-off value (*16*). In our model, if we increased the cut-off value to 1.10, the value used in the original seroprevalence study, the specificity would increase to 98%, which was in good agreement with the results obtained in the validity study, 99% (95% CI 97% to 100%); and, the seroprevalence for May 4-9 period – week 5 of the original seroprevalence study – would decrease to 10.6%, expectedly the same value as the one reported in the original study (*9*, *10*). According to our model, the cut-off value providing the highest accuracy of 98%, is 1.35. This cut-off value corresponds to a maximum number needed to misdiagnose of 52, meaning that on average, one out of 52 people tested is misdiagnosed (either false-positive or false-negative results) (*17*).

One-hundred and thirty-eight of 1432 individuals in the data subset we examined, had SARS-CoV-2 IgG concentrations equal to or more than the cut-off value of 0.90. This translates into an apparent prevalence of 9.6% (95% CI 8.1% to 11.2%). However, because this value is in fact the test-positive rate; it is an unbiased estimate for the true prevalence only if we use a test with 100% sensitivity and specificity (*20*). This apparent prevalence is usually a biased estimate for the true prevalence. Taking into account the test sensitivity and specificity, nonetheless, it is possible to calculate the true prevalence, which is 7.0% (5.4% to 8.6%) in this case. The true prevalence was in good agreement with the estimated prevalence of 7.7% directly obtained from the model, showing the acceptable predictability of the model in estimating the prevalence. This indicates that our model can provide the true not the apparent prevalence (*20*).

The good fit results (*r*^2^ = 0.90), the acceptable agreement between the calculated indices and those obtained from the validity study, and the satisfactory overlap of the ROC curve derived from our model with the one obtained from the validity study, reflect that our educated guess that there should be two subpopulations – one with SARS-CoV-2 IgG in their sera and another without SARS-CoV-2 antibodies in their sera (including those with cross-reacting antibodies with the SARS-CoV-2 IgG) in our test – might be correct. Had the IgG concentrations been measured in samples belonging to the pre-pandemic era, we would have only observed the light gray dashed curve in Figure 3.

The prevalence of SARS-CoV-2 infection as well as the fraction of people with cross-reacting antibodies (resulting in false-positive test results) would affect the interpretation of results (*6*). For example, if the prevalence of the disease is less than the cross-reactivity rate (as happened early in the pandemic), then this method could be problematic as there would be no apparent second peak to be picked up by the proposed algorithm. If the second peak could be identified, the interpretation is straight forward, especially when we examine the probability density functions of the IgG distribution in the two groups. For example, the probability of observing an IgG value of 1.5 (a positive test result) would be 0.432 in those with SARS-CoV-2 IgG compared with 0.024 in those without SARS-CoV-2 IgG (maybe one with cross-reacting antibodies), translating into a likelihood ratio of 18.07. In other words, an IgG concentration of 1.5 is about 18 times more likely to be observed in a person with SARS-CoV-2 IgG (presumably previously infected) as compared with a person with a false-positive test result (maybe one with cross-reacting antibodies). Using the proposed technique, we can calculate the likelihood ratio for each value of SARS-CoV-2 IgG, an index which cannot be readily calculated in validation studies (*24*).

In conclusion, it is possible to derive test performance indices (*e.g.*, sensitivity, specificity, positive and negative predictive values, and positive and negative likelihood ratios), as well as the most appropriate test cut-off value and the prevalence of the condition of interest, without the need for conducting a validity study and comparing the test results against a gold-standard.

## Data Availability

The data that support the findings of this study are available from the *Unité d’Épidémiologie Populationnelle* of the *Hôpitaux Universitaires de Genève* (HUG) researchers, but restrictions apply to the availability of these data, which were used under license for the current study, and so are not publicly available. Data are however available from the authors upon reasonable request and with permission of the HUG researchers.
